# Supplementary material for: Depression, suicidal ideation and suicide risk in German veterinary medical students compared to the German general population
Source: PLoS One. 2022 Aug 17;17(8):e0270912. doi: 10.1371/journal.pone.0270912 (PMC9384977; doi:10.1371/journal.pone.0270912)
Supplement: S1 Table — (DOCX) [file pone.0270912.s001.docx]

| Table 1. Characteristics of participants. | | | | | | | | | | | | | | | | | |
| --- | --- | --- | --- | --- | --- | --- | --- | --- | --- | --- | --- | --- | --- | --- | --- | --- | --- |
|  | | | | | |  | | | | | | | | | | | |
| Veterinary Students | | | | | | German General Population | | | | | | | | | | | |
|  | | | | | | REP_14 angepasst | | | | | | REP_23 angepasst | | | | | |
| Female | | Male | | Total | | Female | | Male | | Total | | Female | | Male | | Total | |
| *N*  828 | %  90.7 | *N*  84 | %  9.2 | *N*  913 | %  100 | *N*  608 | %  55.4 | *N*  489 | %  44.6 | *N*  1097 | %  100 | *N*  579 | %  56.1 | *N*  454 | %  43.9 | *N*  1033 | %  100 |
|  | | | | | | | | | | | | | | | | | |
| Age | | | | | | | | | | | | | | | | | |
| *M* | | | *SD* | | | *M* | | | *SD* | | | *M* | | | *SD* | | |
| 23.57 | | | 3.96 | | | 33.86 | | | 8.20 | | | 32.83 | | | 8.04 | | |
|  | | |  | | |  | | |  | | |  | | |  | | |
| Range 18 – 46^1^ | | | | | | Range 18 - 46 | | | | | | Range 18 - 46 | | | | | |
|  | | | | | |  | | | | | | | | | | | |
|  | | | | | | Female | | | | Male | | | | Total | | | |
|  | | | | | | *N*  2015 | | %  66.2 | | *N*  1027 | | %  33.8 | | *N*  3042 | | %  100 | |
|  | | | | | | Age | | | | | | | | | | | |
|  | | | | | | *M* | | | | | | *SD* | | | | | |
|  | | | | | | 30.51 | | | | | | 8.443 | | | | | |

^1^n = 37 veterinary students did not provide any information about their age

Table 2: Depression, suicidal ideation and suicide risk in veterinary students and the German general population

|  | | **Veterinary Students1** | | | | | |  | **General Population2** | | | | | |  | **Statistical tests Vet vs. general population3** |
| --- | --- | --- | --- | --- | --- | --- | --- | --- | --- | --- | --- | --- | --- | --- | --- | --- |
|  | | **Female** | | **Male** | | **Total** | |  | **Female** | | **Male** | | **Total** | |  |  |
|  | | *n* = 828 | | *n* = 84 | | *N* = 913 | |  | *n* = 605 | | *n* = 489 | | *N* = 1094 | |  |  |
| **Level of Depression** | |  |  |  |  |  |  |  |  |  |  |  |  |  |  |  |
| **PHQ-9** | | *m* = 10.04 | *sd* = 5.99 | *m* = 7.54 | *sd* = 5.76 | *m* = 9.81 | *sd* = 6.01 |  | *m* = 2.37 | *sd* = 3.34 | *m* = 1.66 | *sd* = 2.95 | *m* = 2.05 | *sd* = 3.19 |  |  |
| Severity | Minimal | 164 | 19.9 | 32 | 38.6 | 196 | 21.6 |  | 496 | 82.0 | 433 | 88.5 | 929 | 84.9 |  |  |
| Mild | | 273 | 33.1 | 24 | 28.9 | 297 | 32.7 |  | 88 | 14.5 | 42 | 8.6 | 130 | 11.9 |  |  |
| Moderate | | 188 | 22.8 | 16 | 19.3 | 205 | 22.6 |  | 14 | 2.3 | 8 | 1.6 | 22 | 2.0 |  |  |
| Moderately severe to severe | | 200 | 24.2 | 11 | 13.2 | 211 | 23.3 |  | 7 | 1.2 | 6 | 1.2 | 13 | 1.2 |  |  |
| Total score moderate to severe >=10 | | 388 | 47.0 | 27 | 32.5 | 416 | 45.9 |  | 21 | 3.5 | 14 | 2.8 | 35 | 3.2 |  | OR = 22.076  (CI = 13.873 - 35.131)*** |
| **Suicidal Ideation** | |  | | | | | |  |  | | | | | |  |  |
| **PHQ-9 / Item 9** | | **Female** | | **Male** | | **Total** | |  | **Female** | | **Male** | | **Total** | |  |  |
| Not at all | | 655 | 79.4 | 73 | 86.9 | 729 | 80.1 |  | 572 | 94.5 | 473 | 96.7 | 1045 | 95.5 |  |  |
| Several days | | 121 | 14.7 | 6 | 7.1 | 127 | 14.0 |  | 27 | 4.5 | 14 | 2.9 | 41 | 3.7 |  |  |
| More than half the days | | 35 | 4.2 | 2 | 2.4 | 37 | 4.1 |  | 3 | 0.5 | 1 | 0.2 | 4 | 0.4 |  |  |
| Nearly every day | | 14 | 1.7 | 3 | 3.6 | 17 | 1.9 |  | 3 | 0.5 | 1 | 0.2 | 4 | 0.4 |  |  |
| Sum > 0 | | 170 | 20.6 | 11 | 13.1 | 181 | 19.9 |  | 33 | 5.5 | 16 | 3.3 | 49 | 4.5 |  | OR = 3.960  (CI = 2.542 - 6.168)*** |
| **Suicide Risk** | |  | | | | | |  |  | | | | | |  |  |
| **SBQ-R**^5^ | | **Female** | | **Male** | | **Total** | |  | **Female**  *n* = 578 | | **Male**  *n* = 453 | | **Total**  *N* = 1031 | |  |  |
| >7 (Suicide Risk) | | 191 | 24.4 | 17 | 21.3 | 208 | 24.0 |  | 42 | 7.3 | 26 | 5.7 | 68 | 6.6 |  | OR = 4.166  (CI = 2.829 – 6.135)*** |
| =<7 (none/minimal  Suicide Risk) | | 593 | 75.6 | 63 | 78.8 | 657 | 76.0 |  | 536 | 92.7 | 427 | 94.3 | 963 | 93.4**4** |  |  |

1 Study participants over the age of 46 years were excluded

2 Representative samples from the German general population from 2007 (depression, suicidal ideation) and 2015 (SBQ-R, suicide risk)

3 Binary logistic regression including groups (general population as reference category vs. veterinary students) controlled for age and gender with depression/suicidal ideation/suicide risk as dependent variables, n = 37 veterinary students were excluded from the analysis due to missing data concerning their age

4 One participant with diverse gender was included in the overall analyses (“total”)

5 Suicide Behaviors Questionnaire, maximum score of 18 possible
